# Identification of cuproptosis-related genes in chronic apical periodontitis based on bulk and single-cell RNA sequencing analyses and experimental validation

**DOI:** 10.3389/fimmu.2025.1559220

**Published:** 2025-08-20

**Authors:** Caiyi Zhang, Jie Zhao, Keqing Pan, Lingshuang Liu, Mengyu Jiao, Changqing Yuan, Chunyan Wan

**Affiliations:** Department of Stomatology, the Affiliated Hospital of Qingdao University, Qingdao, China, School of Stomatology, Qingdao University, Qingdao, China

**Keywords:** cuproptosis, chronic apical periodontitis, single-cell RNA sequencing, copper homeostasis imbalance, cell-cell communication

## Abstract

**Background:**

Chronic apical periodontitis (CAP) is a prevalent oral inflammatory disease, yet the complex mechanisms underlying its etiology remain unclear. A recently identified cell death pathway known as cuproptosis may be linked to this condition.

**Methods:**

Differentially expressed cuproptosis-related genes (DE-CRGs) were identified by integrating human CAP dataset (GSE237398) with health control (HC) dataset (GSE223924) from the Gene Expression Omnibus (GEO) database. Subsequently, single-cell RNA sequencing (scRNA-seq) data from clinical samples with CAP (n=3) and HC (n=3) from the GSE171213 dataset were analyzed to assess variations across different cell clusters. The association of CRGs with macrophages and fibroblasts in periodontitis was then explored. Fibroblasts and macrophages were selected for further analysis, which included subset classification, cell-chat analysis, and functional enrichment analysis. Additionally, Receiver Operating Characteristic (ROC) curves were employed to evaluate the discriminatory ability of gene features. Changes in DE-CRGs within the whole periodontitis tissue were confirmed through quantitative real-time PCR (qRT-PCR) and immumohistochemical staining (IHC).

**Results:**

Eight CAP-related DE-CRGs were identified through bulk mRNA sequencing. Numerous interactions among these CRGs were observed, highlighting the complexity of protein-protein interactions. ROC curve analysis demonstrated strong diagnostic potential for these genes. ScRNA-seq sequencing revealed significant alterations in CRGs within fibroblasts and macrophages, along with close intercellular communication between these cell clusters. qRT-PCR and IHC analysis of clinical samples further confirmed DE-CRGs expression in CAP.

**Conclusion:**

These findings suggest that CRGs are closely associated with the COL4A1-Fibro and APOE-Macro intercellular interactions, which may facilitate the occurrence and progression of cuproptosis in chronic apical periodontitis.

## 1 Introduction

Apical periodontitis (AP) refers to an inflammatory reaction occurring in the tissues surrounding the apex of the tooth, commonly resulting from bacterial infection in the root canal. AP is typically diagnosed based on symptoms, radiographic findings, and pulp vitality tests. Reports indicate that the prevalence of AP is 52%, with a higher incidence observed in individuals with systemic conditions (1). The disease can progress in either an acute or chronic manner, often presenting as a chronic asymptomatic illness. Chronic apical periodontitis (CAP) is characterized by an exaggerated immune response to persistent irritants within the root canal, which primarily leads to the destruction of periapical tissues, resorption of alveolar bone, and the formation of inflammatory granulation tissue (2). Throughout this process, complex regulatory mechanisms govern the activity of host effector cells and signaling molecules during interactions with pathogenic microbes are involved (3).

In recent years, cuproptosis has been identified and implicated in various diseases. This newly defined form of programmed cell death is related to mitochondrial respiration and is distinct from apoptosis, pyroptosis, ferroptosis, and necrosis (4, 5). Copper, a trace element in the human body, has been strongly associated with various signaling pathways. Excess intracellular copper induces the aggregation of lipoylated dihydrolipoamide S-acetyltransferase (DLAT), which is linked to the mitochondrial tricarboxylic acid (TCA) cycle. This aggregation results in proteotoxic stress and leads to a novel form of cell death termed cuproptosis (6). Studies have demonstrated that cuproptosis plays a significant role in the pathogenesis of multiple diseases, including cancer (7), rheumatoid arthritis (8), and cardiovascular diseases (9). Additionally, it has been reported to be associated with the development of osteoporosis, suggesting that cuproptosis may serve as a potential target for osteoporosis treatment (10). Furthermore, in oral diseases, cuproptosis has been shown to be a crucial factor in the pathophysiological process of periodontitis (11, 12). Pulpal infection spreads through the apical foramen into the periapical tissues, ultimately leading to the development of AP (13). It has been demonstrated that cuproptosis exacerbates the progression of pulpitis by suppressing the pentose phosphate pathway (14). However, the role of cuproptosis in CAP and its underlying molecular mechanisms remains to be investigated.

The current study aimed to investigate the relationship of copper-dependent cell death with chronic apical periodontitis. Cuproptosis-related genes (CRGs) are implicated in this form of cell death. In our research, we screened 19 CRGs using conventional bulk sequencing and single-cell RNA sequencing (scRNA-seq) to detect expression changes of relevant genes across different cell clusters in chronic apical periodontitis. Additionally, we confirmed the alteration of key genes in human periapical tissues using quantitative real-time PCR (RT-PCR) and immumohistochemical staining (IHC). Our study provides insights into the characteristic imbalance of copper homeostasis in CAP, enhancing our understanding of its pathogenesis and contributing to the identification of effective therapeutic strategies.

## 2 Materials and methods

### 2.1 Bulk RNA sequencing

#### 2.1.1 Data acquisition

We retrieved two microarray datasets from the Gene Expression Omnibus (GEO) database (http://www.ncbi.nlm.nih.gov/geo/): GSE237398 (containing 10 CAP tissue samples) (15) and GSE223924 (comprising 10 healthy periodontal tissue samples) (16) from the Gene Expression Omnibus (GEO) database (http://www.ncbi.nlm.nih.gov/geo/). The 19 CRGs (NFE2L2, SLC31A1, FDX1, LIAS, DLD, DLAT, DBT, NLRP3, LIPT1, PDHA1, PDHB, ATP7A, ATP7B, GLS, MTF1, CDKN2A, SLC25A3, GCSH, and DLST) used in our study were based on previous studies (4, 17).

#### 2.1.2 Determination of differentially expressed genes

Prior to conducting differential gene expression analysis, read counts for each sequenced library were adjusted using the edgeR program package through a single scaling normalization factor. The DEGs between healthy tissues and those affected by CAP were identified using the the edgeR package (3.40.2) (18) and presented in heat maps and volcano plots. In the current analysis, DEGs were identified using a significance threshold of adjusted *p* < 0.05 combined with an absolute log2 fold change (log2FC) > 1.

#### 2.1.3 Protein-protein interaction network construction and module analysis

We utilized the STRING database (http://string-db.org/) (19), which is known for predicting Protein-Protein Interactions, to construct a PPI network of the CRGs. An interaction was deemed significant when the composite score exceeded 0.4.

#### 2.1.4 Construction of the prognostic model

To evaluate the diagnostic performance of the predictive model, a Receiver Operating Characteristic (ROC) curve was generated. The area under the curve (AUC) was calculated to quantify the model’s discriminative ability. AUC values approaching 1 indicate superior diagnostic performance, while values near 0.5 suggest that the model performs no better than random chance.

### 2.2 Single-cell RNA sequencing

#### 2.2.1 Ethics and clinical sample collection

Three periapical lesion samples were collected from extracted teeth using a sterile knife and were immediately transported to the laboratory on dry ice. Sample A was obtained from a 57-year-old male, Sample B from a 25-year-old female, and Sample C from a 51-year-old female. The inclusion criteria were as followed: (1) a confirmed diagnosis of chronic apical periodontitis; (2) systemically healthy individuals without diabetes mellitus, immunodeficiency, or malignancies; (3) no use of antibiotics or anti-inflammatory medications within three months prior to sampling; and (4) non-restorable affected teeth. The exclusion criteria were: (1) the presence of systemic diseases such as diabetes mellitus, immunodeficiency, or malignancies; (2) concurrent acute pulpitis or acute apical inflammation; and (3) pregnant or lactating women. Informed consent was obtained from all patients. This study was approved by the Ethics Committee of The Affiliated Hospital of Qingdao University (No. QYFY WZLL 28579) and was conducted in accordance with the principles outlined in the Declaration of Helsinki. The procedure for preparing single-cell suspensions was based on a previous study (20).

#### 2.2.2 scRNA-seq

The cell suspension was loaded into Chromium microfluidic chips using 3’ chemistry (v2) and barcoded with a 10× Chromium Controller (10× Genomics). RNA from the barcoded cells was subsequently reverse-transcribed, and sequencing libraries were constructed using reagents from a Chromium Single Cell 3’ v2 reagent kit (10× Genomics), following the manufacturer’s instructions. Sequencing was conducted with Illumina platforms in accordance with the manufacturer’s guidelines (Illumina).

#### 2.2.3 Two atlas cell datasets integration and single-cell sequencing data analysis

The scRNA-seq data from three healthy control (HC) samples, obtained from clinically healthy periodontal tissues, were sourced from the GSE171213 dataset (GSM5220921, GSM5220922, and GSM5220923) (21). The HC datasets were integrated with three datasets from chronic CAP cases using the Harmony package (22). Subsequently, principal component analysis (PCA) was performed with 30 dimensions, followed by clustering using the FindClusters function at a resolution of 0.6 to explore cell heterogeneity. A list of marker genes from the literature and the CellMarker 2.0 database (23) was utilized to annotate the cell subpopolations. Differential expression analysis conducted with the limma package led to the identification of differentially expressed genes, defined by an adjusted *p*-value < 0.05 and log2FC > 0.25. Additionally, the R package CellChat was employed to assess cell–cell interactions based on existing databases.

#### 2.2.4 Functional analysis of DE-CRGs in fibroblast and macrophage

To validate the potential function of the DE-CRGs, we conducted Gene Ontology (GO) (24) and Kyoto Encyclopedia of Genes and Genomes (KEGG) (25) enrichment analyses. The GO enrichment analysis of differentially expressed genes was performed using the clusterProfiler R package (4.6.2) (26), which corrected for gene length bias. GO terms with a corrected *p*-value of less than 0.05 were considered significantly enriched functions within the gene set. The KEGG database serves as a resource for understanding high-level functions and utilities of biological systems, including cells, organisms, and ecosystems, derived from molecular-level information, particularly large-scale molecular datasets generated by genome sequencing and other high-throughput experimental technologies (http://www.genome.jp/kegg/). We utilized the clusterProfiler R package to assess the statistical enrichment of differentially expressed genes in KEGG pathways.

### 2.3 Experimental verification

#### 2.3.1 Real-time quantitative PCR

We collected three samples of periapical tissue and three healthy tissues for qRT-PCR. The criteria including X-ray results and operative exploration was described in previous study (27). Total RNA was extracted using Steadypure Total RNA Extraction Reagent (AG, Hunan, China). First-strand cDNA synthesis was performed using the Reverse Transcription System (AG, Hunan, China) according to the manufacturer’s instructions. qRT-PCR was performed with SYBR Green Premix *Pro Taq* HS qPCR Kit (AG, Hunan, China). The following genes were quantified: (NFE2L2, NLRP3, DLST, ATP7A, ATP7B, GLS, SLC25A3, and GCSH). GAPDH was used as the internal normalization control. Primer sequences are shown in **Table 1**. The expression of each gene was calculated using the 2^-ΔΔCT^ methods. The gene expression ratio was shown as mean ± standard deviation from three independent experiments.

**Table 1:** Oligonucleotide primer sequences used in qRT-PCR.

Gene	Sequence (5’-3’)	
NFE2L2	Forward	TCAGCGACGGAAAGAGTATGA
	Reverse	CCACTGGTTTCTGACTGGATGT
NLRP3	Forward	CGTGAGTCCCATTAAGATGGAGT
	Reverse	CCCGACAGTGGATATAGAACAGA
DLST	Forward	GGTTCCATCACCAGCAAA
	Reverse	AGTCCCAATCCCAAGAGG
ATP7A	Forward	TGTGTGCAGTCTATTGAGGGT
	Reverse	TGACAAGGTAGCATCAAATCCC
ATP7B	Forward	GGCCGTCATCACTTATCAGCC
	Reverse	GGGAGCCACTTTGCTCTTGA
GLS	Forward	ATTCAGTCCCGATTTGTGGGG
	Reverse	AGAAGGGAACTTTGGTATCTCCA
SLC25A3	Forward	TGGTGTTCGTGGTTTGGCTAA
	Reverse	GATGTGCGCCAGAGATAAGTATT
GCSH	Forward	GTCTCCCTGAAGTTGGGACA
	Reverse	TCTGAAGGGTTACTCAGTGTCA

#### 2.3.2 Immumohistochemical staining

Human tissues were fixed in 4% paraformaldehyde and subsequently embedded in paraffin. The sections were deparaffinized in xylene and rehydrated through a series of graded ethanol solutions. To block non-specific binding sites, 3% BSA was applied. The slides were incubated overnight at 4°C with primary antibodies against ATP7A (1:200, BIOSS), ATP7B (1:200, PTGLAB), NFE2L2 (1:200, BIOSS), and SLC25A3 (1:200, BIOSS). Following incubation, sections were washed with PBS and incubated with HRP-conjugated secondary antibodies for 30 minutes. Signal detection was carried out using DAB chromogen, followed by hematoxylin counterstaining. The slides were dehydrated, mounted, and examined under a light microscope. For each marker, staining intensity and the percentage of positive cells were scored semi-quantitatively in five randomly selected high-power fields per slide.

### 2.4 Statistical analysis

GraphPad Prism 9.5 and Seurat R v4.3.0.1 (https://www.R-project.org) were used for data processing nd analyses. Differences between groups were analyzed by Student’s t-test. All statistical p-values were two-tailed, and *p* < 0.05 was considered statistically significant. *p < 0.05, **p < 0.01, ***p < 0.005.

## 3 Results

### 3.1 Identifification of cuproptosis in chronic apical periodontitis

**Figure 1:**
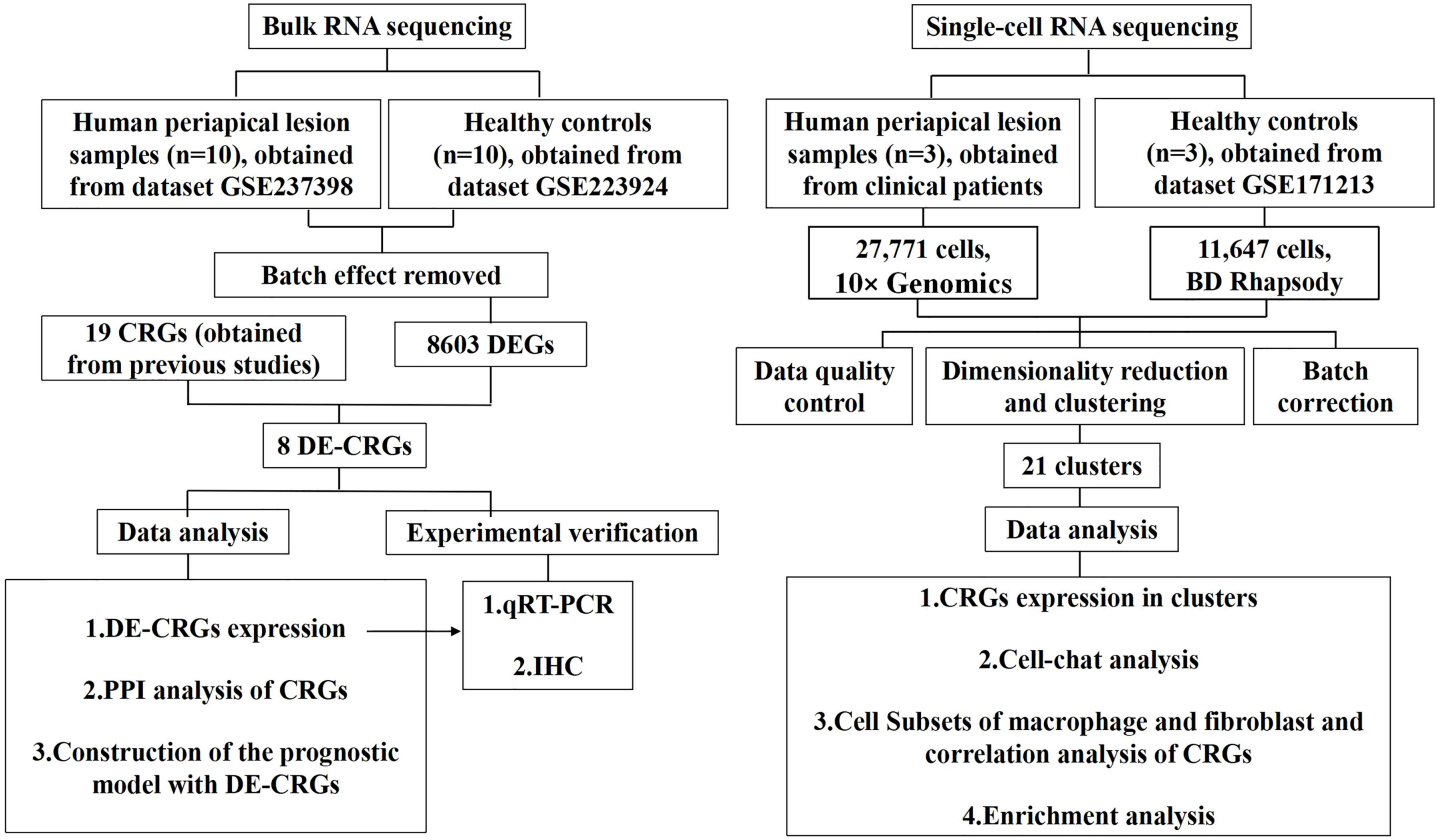
Flowchart of the whole study strategy on bioinformatic data. CRGs, cuproptosis-related genes; DEGs, differentially expressed genes; DE-CRGs, differentially expressed cuproptosis-related genes; PPI, protein-protein interaction.

**Figure 2:**
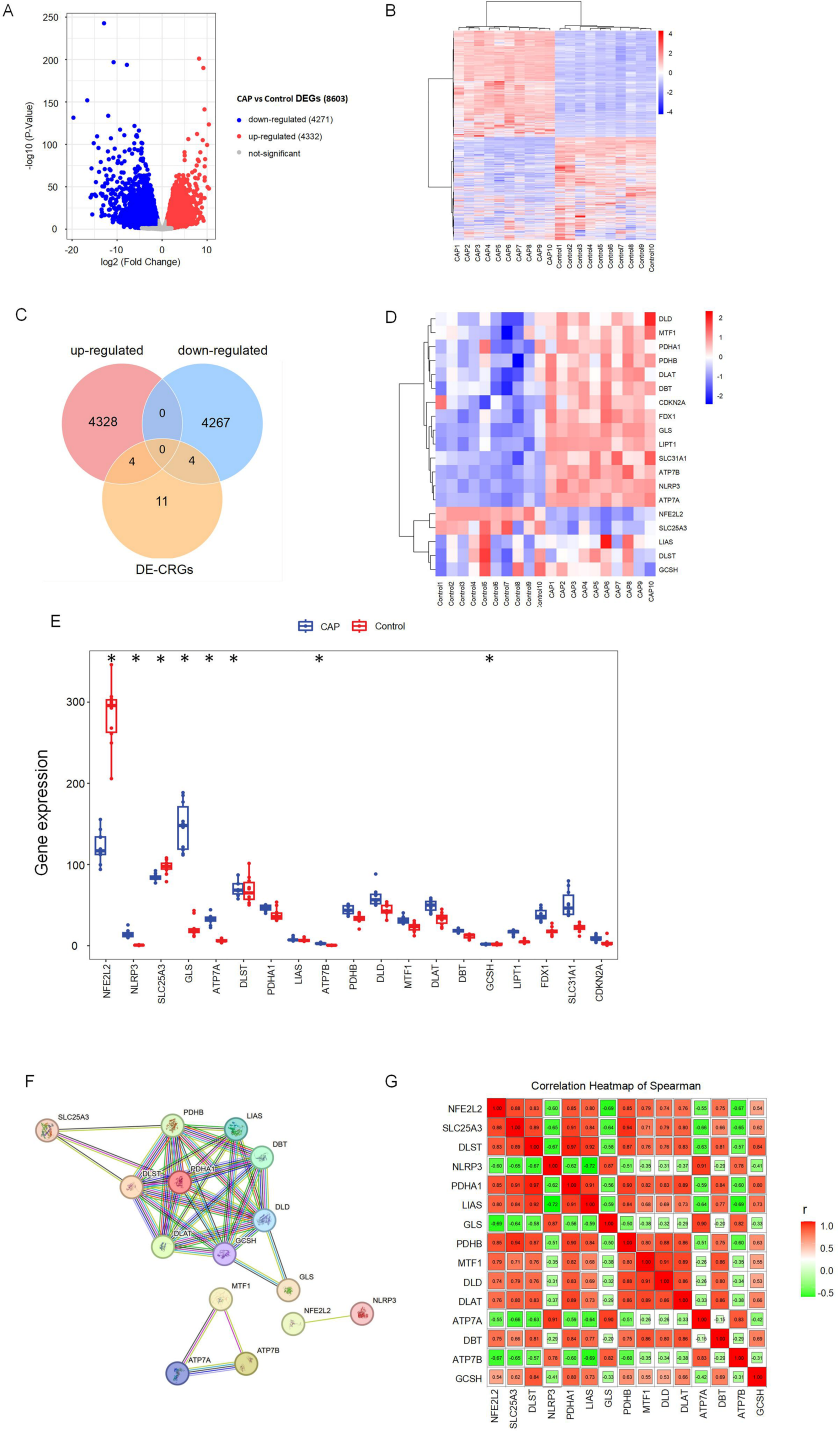
Identification of differentially expressed cuproptosis-related genes (DE-CRGs) in CAP and HC. **(A)** Volcano plots of DEGs, and adj. *p* value < 0.05, the up-expressed mRNAs are shown in red and the down-expressed mRNAs are shown in blue, the X-axis represents log2FC, the Y-axis represents represents -log10 (adj. *p* value). **(B)** Clustered heatmap of DEGs. **(C)** Venn diagram, determination of 8 DE-CRGs. **(D)** Clustered heatmap of DE-CRGs. **(E)** The bar graph shows the mRNA expression of 8 DE-CRGs between control group and CAP group. **(F)** Protein-protein interaction (PPI) network of the CRGs. **(G)** Correlations of the CRGs in the CAP samples Red, positive correlation; Green, negative correlation. The color depth and the size reflect the strength of the relevance. *p < 0.05.

**Figure 1** illustrates the overall study design framework. Ten samples from the GSE223924 dataset (GSM7006826, GSM7006827, GSM7006828, GSM7006829, GSM7006830, GSM7006831, GSM7006832, GSM7006833, GSM7006834, and GSM7006835) were selected as the control group, while ten samples from the GSE237398 dataset (GSM7610850, GSM7610851, GSM7610852, GSM7610853, GSM7610854, GSM7610855, GSM7610856, GSM7610857, GSM7610858, and GSM7610859) were designated as the apical periodontitis group. Utilizing the edgeR package, we identified a total of 8603 DEGs, which included 4332 upregulated genes and 4,271 downregulated genes (**Figure 2A**). A heatmap of differentially expressed genes revealed distinct expression patterns between the treatment and control groups, highlighting clusters of upregulated and downregulated genes (**Figure 2B**). Further details regarding the DEGs can be found in **Supplementary Table 1**.

Nineteen CRGs were mapped to the DEG set, resulting in the identification of eight DE-CRGs associated with apical periodontal tissue. A Venn diagram was generated to illustrate the DE-CRGs using the VennDiagram R package (28) (**Figure 2C**). Among these, four CRGs were found to be down-regulated in chronic apical periodontitis (CAP), namely Nuclear Factor Erythroid 2-Related Factor 2 (NFE2L2), Dihydrolipoamide S-Succinyltransferase (DLST), Solute Carrier Family 25 Member (SLC25A3), and Glycine Cleavage H Protein (GCSH). In contrast, four CRGs were up-regulated in CAP, including NLR Family Pyrin Domain Containing 3 (NLRP3), Copper Transporting ATPases (ATP7A, ATP7B), and Glutaminase (GLS) (**Figures 2D, E**).

### 3.2 PPI analysis of CRGs

To identify protein relationships, we constructed a PPI network of CRGs. The results revealed multiple interactions among the CRGs, highlighting the complexity of protein interactions. Specifically, ATP7A, ATP7B, and MTF1 exhibited significant protein-protein interactions, while NFE2L2 and NLRP3 also demonstrated interactions. Furthermore, the remaining 14 CRGs displayed various protein-protein interactions (**Figures 2F, G**). These findings suggest a close relationship between cuproptosis and apical periodontitis.

### 3.3 scRNA-seq and cellular constitution of human apical periodontal tissues

To preliminarily investigate the composition of cell populations in human apical periodontal tissues, we conducted scRNA-seq analysis on human periapical tissues from three healthy controls and three patients with CAP. Following standard data processing and quality control procedures, we obtained single-cell transcriptomes from a total of 39,418 single cells, comprising 11,647 cells from healthy controls and 27,771 cells from patients with CAP.

**Figure 3:**
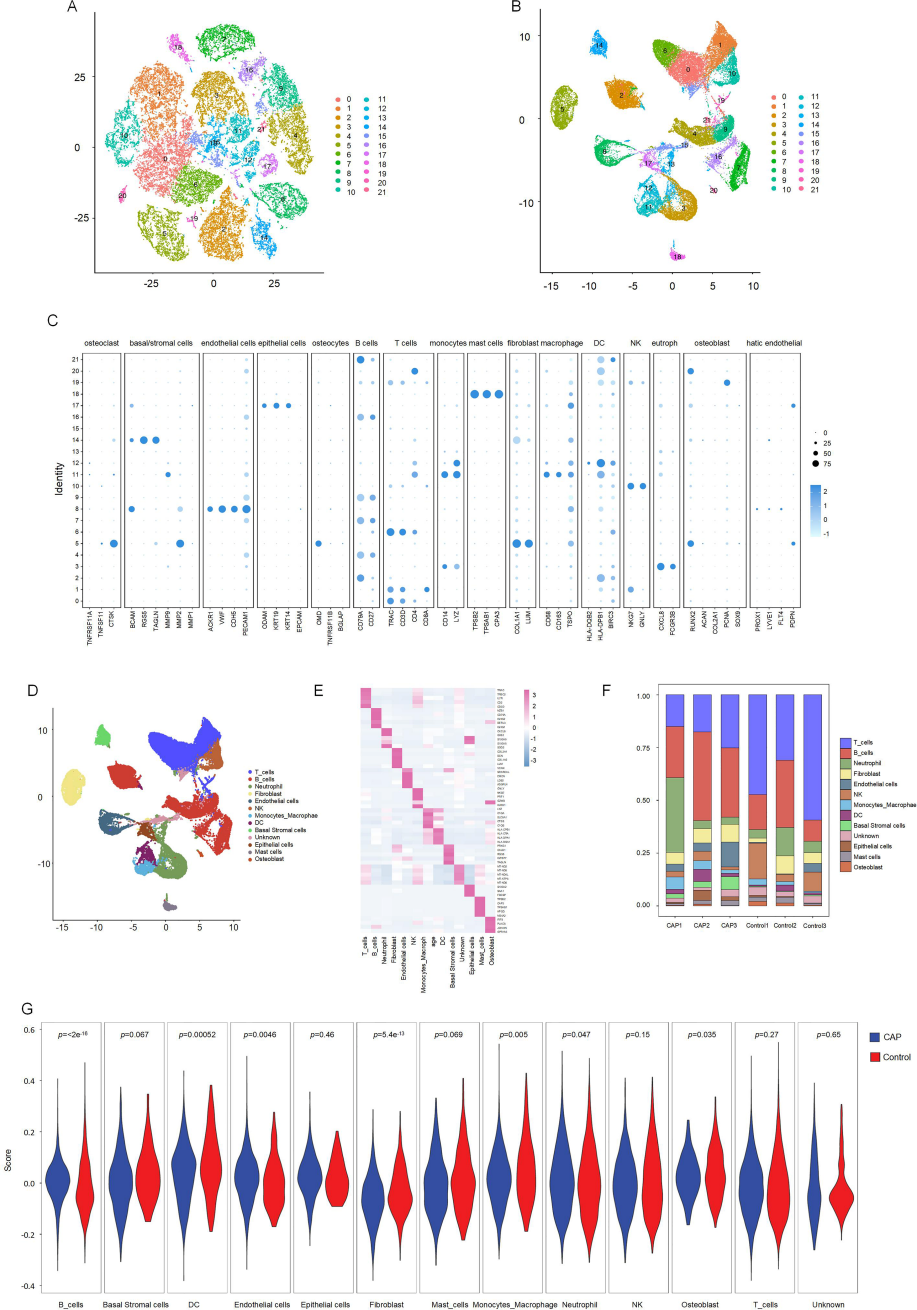
Clustering and differential analysis of scRNA-seq data. **(A, B)** Cells in scRNA-seq were classified into 21 clusters by dimensional reduction and clustering analysis. **(C)** Marker gene expression in each cluster. **(D)** The UMAP diagram diagram shows the distribution of the 13 major cell types in each sample. **(E)** A heatmap showing the top 5 differentially expressed genes in each cell type. **(F)** Histogram overlays display the proportion of cell types in each sample. **(G)** The expression of CRGs in each cell type.

T-SNE and UMAP dimensionality reduction techniques were employed for visualization. As illustrated in **Figures 3A** and **3B**, the cells were classified into 21 distinct clusters. Utilizing the identified marker genes, these clusters were subsequently annotated into 12 major cell types: T lymphocytes, B lymphocytes, neutrophils, fibroblasts, endothelial cells, natural killer (NK) cells, monocytes-macrophages, dendritic cells (DC), basal cells, epithelial cells, mast cells, and osteoblasts (**Figure 3C**). Each cluster was annotated based on the principal markers, and the expression profiles of the representative marker genes across the cell populations were demonstrated. (**Figures 3D, E**, **Supplementary Figure 1**).

We subsequently compared the proportions of each cell cluster between the CAP and HC samples (**Figure 3F**). Our analysis revealed significant reductions in the fractions of T cell and NK clusters within the CAP group relative to the HC group. Notably, the abundance of B cells was slightly elevated in the CAP group compared to the HC group. Additionally, the percentages of endothelial cells, monocytes-macrophages, and basal cells were markedly increased in the CAP group when compared to the HC group.

### 3.4 The interaction between macrophages and fibroblasts represents a potential mechanism regulating cuproptosis in CAP

To investigate the expression changes of CRGs across various cell types within apical periodontal tissue and healthy tissue, we analyzed the scRNA-seq data. The expression levels of CRGs in B lymphocytes, neutrophils, fibroblasts, endothelial cells, monocyte-macrophages, DC, basal cells and mast cells were significantly different between the CAP group and control group (*p* < 0.05) (**Figure 3G**). Featureplot heatmap of each DE-CRGs were demonstrated in **Supplementary Figure 2**.

**Figure 4:**
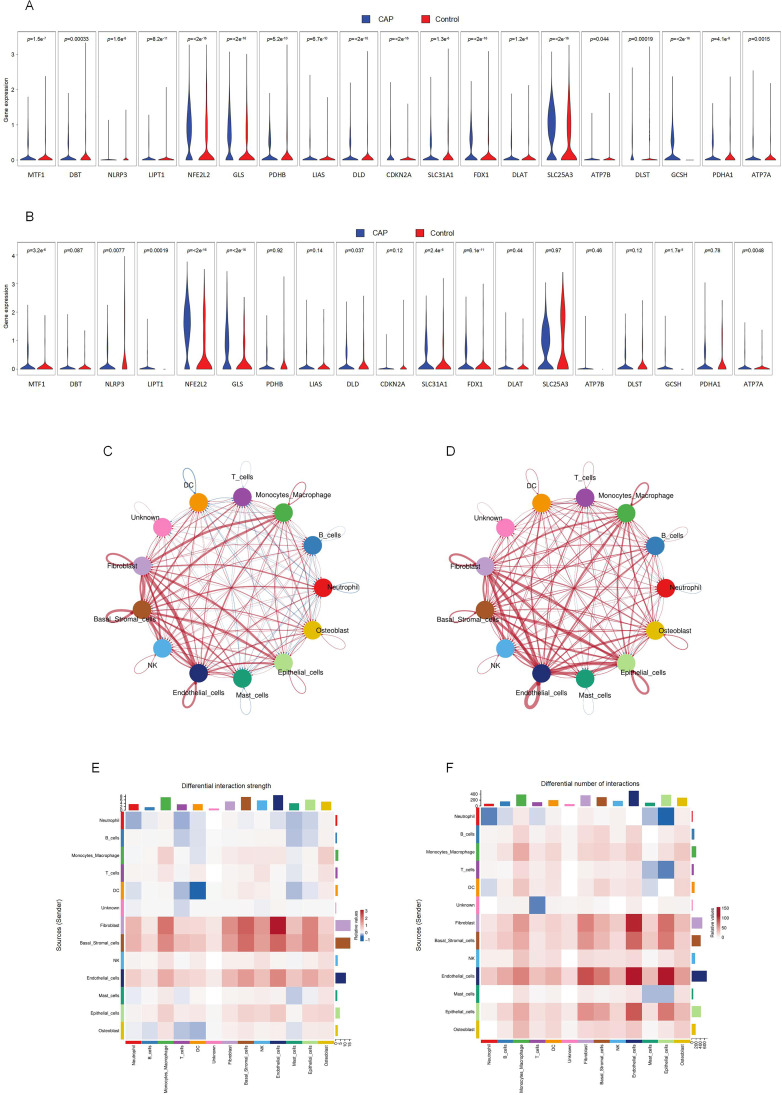
Intercellular communication between clusters and CRGs expression in fibroblasts and macrophages. **(A)** The expression of CRGs in fibroblasts. **(B)** The expression of CRGs in macrophage. **(C)** Differential interaction strength between the clusters by CellChat. Red line represents the CAP group, Blue line represents the control group. **(D)** Differential interaction number between the clusters by CellChat. Red line represents the CAP group, Blue line represents the control group. **(E)** The heatmap shows the differential interaction strength. **(F)** The heatmap shows the differential interaction number.

Significant variations in the expression of CRGs were observed in 2 cell clusters: fibroblast-CAP and macrophage-CAP. To further elucidate the relationship between CRGs and apical periodontiti s, we compared the expression of CRGs in fibroblast and macrophage cell clusters between the CAP and healthy control groups. The expression levels of CRGs in these two cell types are depicted in **Figures 4A** and **4B**. Most CRGs exhibited differential expression, with the exception of DBT, PDHB, LIAS, CDKN2A, DLAT, SLC25A3, ATP7B, DLST, and PDHA1 in macrophages (*p* < 0.05). We utilized CellChat (29), a tool that employs a database of ligand-receptor interactions to analyze cell-cell communication from scRNA-seq data, to identify noteworthy cell subsets. Our findings revealed significant differences in cell-cell interactions between the control and CAP samples, with an increased number of interactions observed in the CAP group (**Figures 4C, D**). Notably, the intensity of cell-cell interactions between fibroblasts and macrophages was significantly enhanced in the CAP group (**Figures 4E, F**).

### 3.5 Cell subsets of macrophage and fibroblast and correlation analysis of CRGs

**Figure 5:**
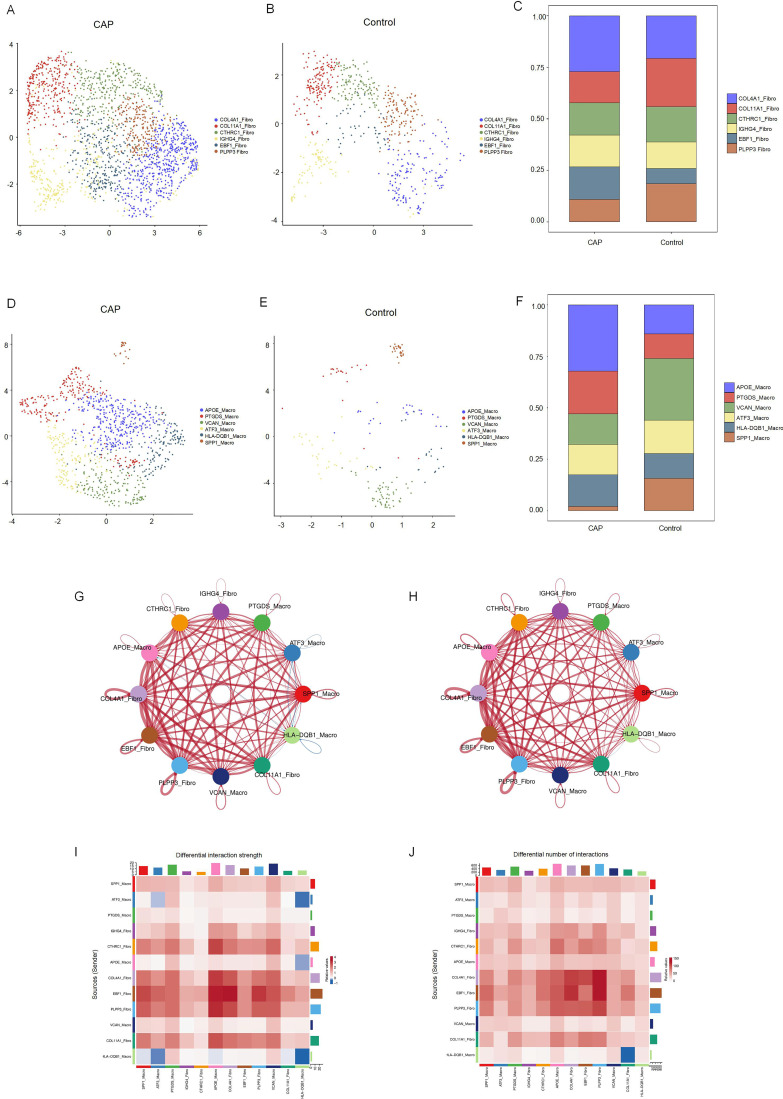
Clustering subtypes and Intercellular communication of fibroblasts and macrophages in scRNA-seq data. **(A)** The UMAP diagram shows the fibroblast cells were classified into 6 clusters using dimensional reduction and clustering analysis in CAP group. **(B)** The UMAP diagram shows the fibroblast cells were classified into 6 clusters using dimensional reduction and clustering analysis in control group. **(C)** Histogram overlays display the proportion of cell clusters in CAP and control group. **(D)** The UMAP diagram shows the macrophage cells were classified into 6 clusters using dimensional reduction and clustering analysis in CAP group. **(E)** The UMAP diagram shows the macrophage cells were classified into 6 clusters using dimensional reduction and clustering analysis in control group. **(F)** Histogram overlays display the proportion of cell clusters in CAP and control group. **(G)** Differential interaction strength between the clusters by CellChat. Red line represents the CAP group, Blue line represents the control group. **(H)** Differential interaction number between the clusters by CellChat. Red line represents the CAP group, Blue line represents the control group. **(I)** The heatmap shows the differential interaction strength. **(J)** The heatmap shows the differential interaction number.

We identified six distinct subclusters of fibroblasts, each characterized by multiple differentially expressed genes (**Figures 5A–C**). These subclusters include COLA1-Fibro, COL11A1-Fibro, CTHRC1-Fibro, IGHG4-Fibro, EBF1-Fibro, and PLPP1-Fibro. In chronic apical periodontal tissues, the fibroblast subpopulations COL4A1-Fibro and EBF1-Fibro were found to occupy a more dominant position. Furthermore, we identified six subclusters of macrophages, each characterized by multiple differentially expressed genes (**Figures 5D–F**). These subclusters are APOE-Macro, PTGDS-Macro, VCAN-Macro, ATF3-Macro, HLA-DQB1-Macro, and SPP1-Macro. During the inflammatory cell infiltration associated with chronic apical periodontitis, the macrophage subpopulation APOE-Macro became more predominant. We conducted cell-chat analysis on the subpopulations of fibroblasts and macrophages, revealing that the intensity of cell-cell interactions among APOE-Macro, PTGDS-Macro, VCAN-Macro, SPP1-Macro, and COLA1-Fibro was significantly enhanced in the CAP group (**Figures 5G–J**). Moreover, ligand-receptor pair analysis indicated that various signaling pathways were involved in these intercellular interactions (**Supplementary Figure 3**).

**Figure 6:**
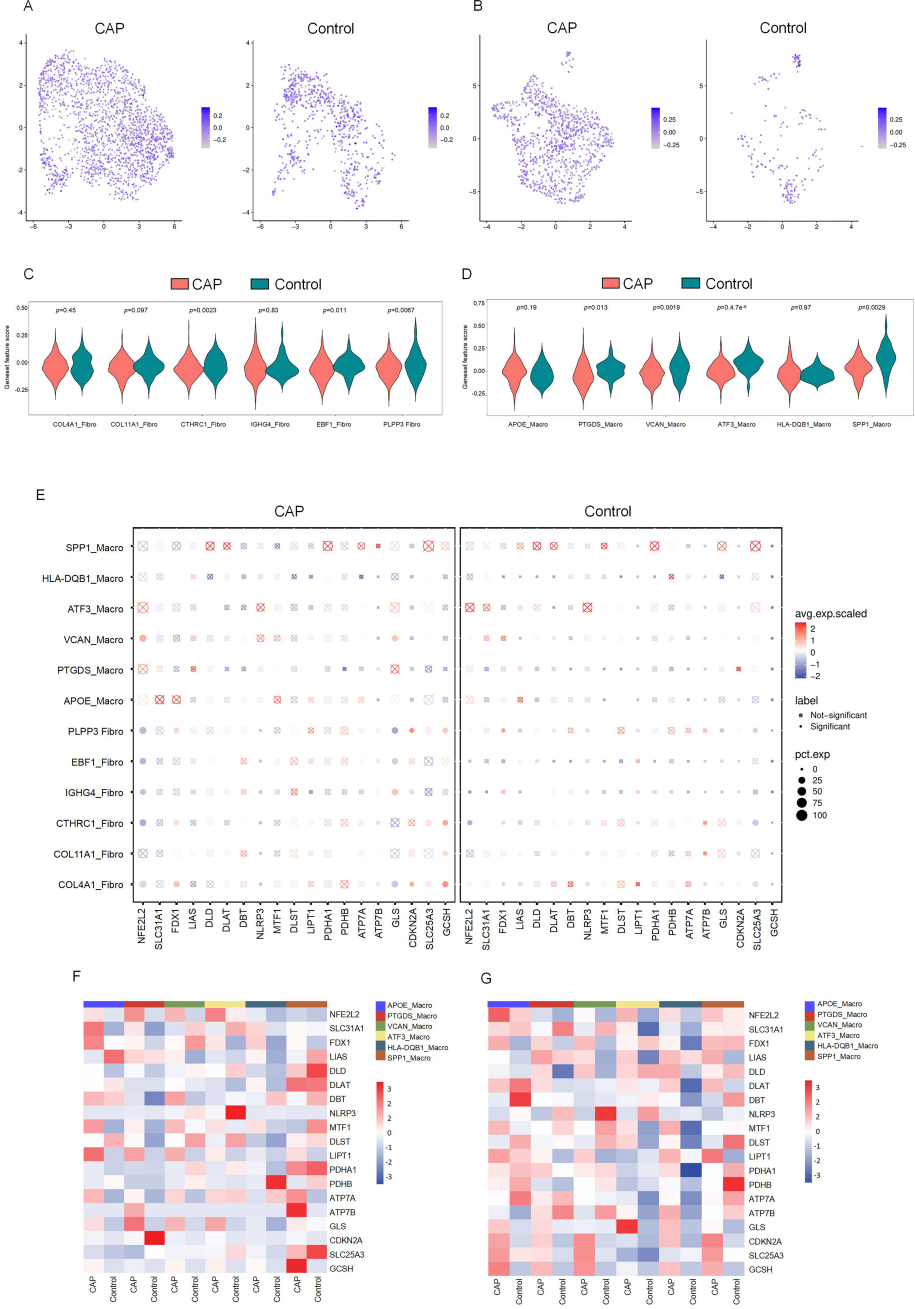
The CRGs expression in cell subsets of fibroblasts and macrophages. **(A)** Featureplot heatmap of CRGs between CAP and Control in fibroblasts. **(B)** Featureplot heatmap between CRGs in CAP and Control in macrophages. **(C)** Violin plots showing the expression of CRGs between fibroblast cell subsets. **(D)** Violin plots showing the expression of CRGs between macrophage cell subsets. **(E)** The bubbleplot of cell subsets. **(F)** The heatmap shows the differential expression of CRGs in macrophage cell subsets. **(G)** The heatmap shows the differential expression of CRGs in fibroblast cell subsets.

The relationship between DE-CRGs and the fibroblast and macrophage subpopulations in CAP was analyzed to further investigate the potential mechanisms by which CRGs influence the progression of CAP. The expression of CRGs in fibroblast and macrophage cell subsets within the CAP and control groups is depicted in **Figures 6A** and **6B**. We found that the cuproptosis scores of CTHRC1-Fibro, EBF1-Fibro, PLPP3-Fibro, PTGDS-Macro, VCAN-Macro, ATF3-Macro, and SPP1-Macro exhibited significant differences (*p* < 0.05), highlighting a close relationship between these cell subsets and cuproptosis (**Figures 6C, D**). Several CRGs were strongly correlated with fibroblast and macrophage cell subsets. For example, NFE2L2 demonstrated a distinct positive correlation with VCAN-Macro, but a negative correlation with fibroblast subsets, except for COL11A1. FDX1 showed a positive correlation with COL4A1-Fibro, CTHRC1-Fibro, and PLPP3-Fibro. GLS exhibited a positive correlation with IGHG4-Fibro and VCAN-Macro, but a negative correlation with PLPP3-Fibro, EBF1-Fibro, and COL4A1-Fibro. CDKN2A was positively correlated with both PLPP3-Fibro and COL4A1-Fibro. SLC25A3 and GCSH demonstrated positive correlations with PLPP3-Fibro, CTHRC1-Fibro, and COL4A1-Fibro. Additionally, GCSH was also positively associated with COL11A1-Fibro (**Figures 6E–G**). These findings indicate that CRGs are generally upregulated across different cell clusters, exhibiting significant variations between macrophage and fibroblast populations, which contrasts with the results of the differential expression analysis of CRGs in the overall tissue.

### 3.6 Enrichment analysis of fibroblast and macrophage cell subsets in CAP

We conducted GO and KEGG enrichment analyses to further elucidate the potential functional roles of fibroblast and macrophage cell subsets. The results indicated that, within the CAP group, the fibroblast cell subsets exhibited significantly higher enrichment levels related to organelle and mitochondrial inner membrane functions, cell adhesion, neuron projection development, and kinase activity. In contrast, the primary functions of the macrophage cell subsets were associated with GTPase activator activity, nucleoside-triphosphatase regulator activity, structural molecule activity, proton-transporting ATPase, and ribosome-related functions (**Supplementary Figures 4, 5**).

### 3.7 Experimental verification

**Figure 7:**
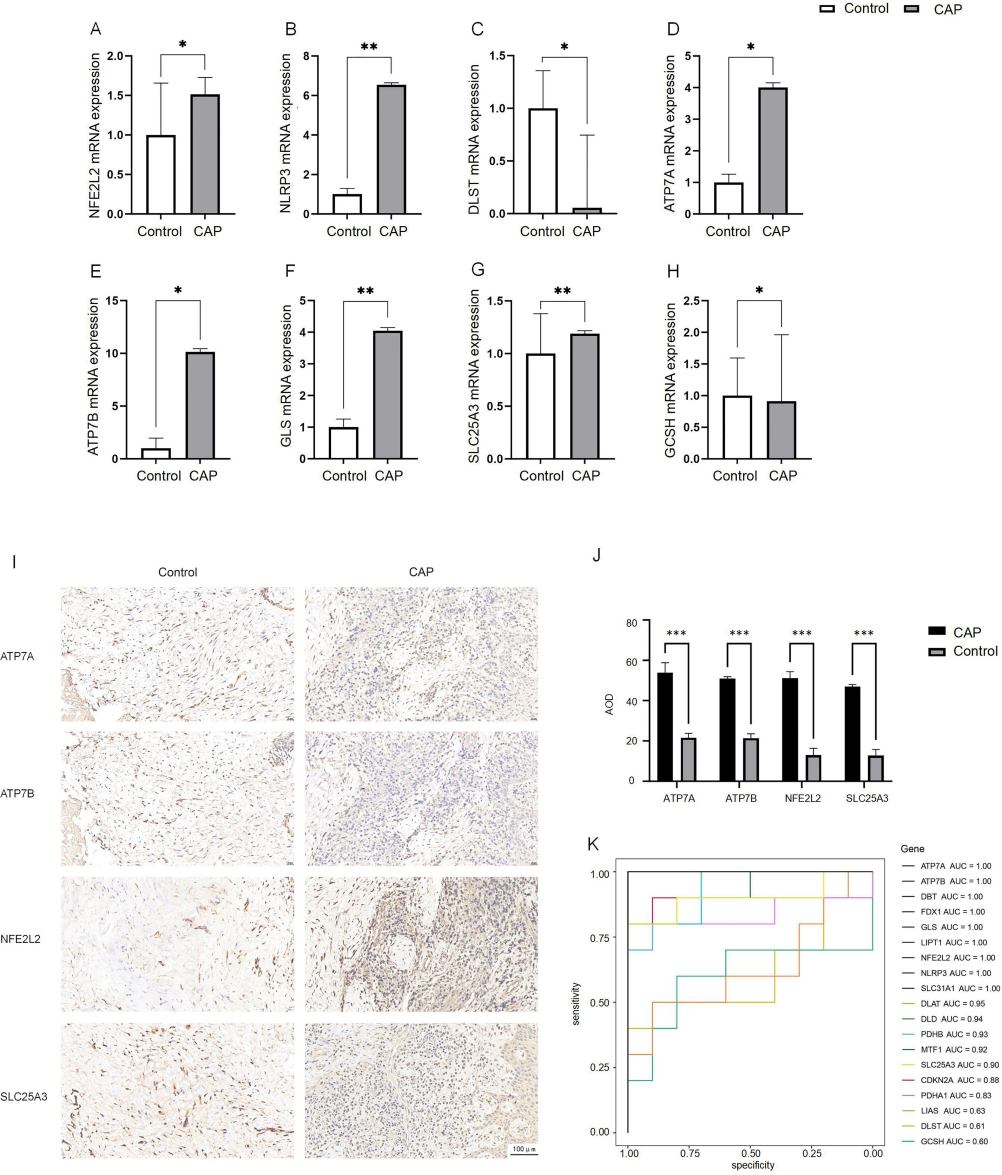
Expression analysis of CRGs in CAP and control.**(A–H)** Expression of 8 DE-CRGs in human healthy control tissue (n = 5) and CAP tissue (n = 5) evaluated by qRT-PCR. **(I, J)** The expression of ATP7A, ATP7B, NFE2L2 and SLC25A3 in human healthy control tissue (n = 5) and CAP tissue (n = 5) evaluated by IHC. **(K)** ROC curve of the19 CRGs. *p < 0.05, **p < 0.01, ***p < 0.005.

To confirm the differences in differentially expressed DE-CRGs in whole tissue, we conducted qRT-PCR to evaluate the expression levels of DE-CRG mRNA in human chronic apical periodontitis tissues compared to healthy tissues. With the exception of the NFE2L2 and SLC25A3 genes, the mRNA expression levels of DE-CRGs in human apical periodontal tissue were consistent with the findings from our bioinformatic analysis. Notably, the mRNA expression levels of NFE2L2 and SLC25A3 were found to be upregulated in CAP tissues (**Figures 7A–H**).

We employed IHC staining to validate the protein expression of four candidate genes: ATP7A and ATP7B, which showed significant differential expression in qPCR analysis, as well as NFE2L2 and SLC25A3, whose bioinformatics predictions conflicted with initial experimental observations. Notably, while bioinformatics analysis predicted downregulation of NFE2L2 and SLC25A3, immunohistochemical results demonstrated significantly increased positive signal areas for both proteins, consistent with qPCR findings. The positive signals were predominantly localized in the nucleus, exhibiting either diffuse or focal distribution patterns (**Figure 7I**). The average optical density (AOD) of the IHC results was analyzed. The AOD values of ATP7A, ATP7B, NFE2L2, and SLC25A3 were significantly higher in the CAP group compared to the HC group (*p* < 0.005) (**Figure 7J**).

### 3.8 Construction of the prognostic model with CRGs

To evaluate the diagnostic value of CRGs in CAP, we performed a univariate logistic regression analysis for each gene. The results indicated that CRGs demonstrated strong diagnostic performance in the training set except DLST, GCSH or LIAS (AUC > 0.8, **Figure 7K**). These findings suggest that the cuproptosis model should be considered in the diagnosis of CAP.

## 4 Discussion

CAP is a prevalent oral disease characterized by periodontal inflammation resulting from an odontogenic infection, which ultimately leads to bone loss (30). The immune-inflammatory response in AP is a complex and dynamic process (31). Although nonsurgical endodontic treatment is the recommended therapeutic approach for AP and demonstrates a high success rate, there are instances where periapical lesions persist despite standard endodontic interventions. A deeper understanding of the inflammatory microenvironment associated with AP could facilitate the development of adjunct therapies aimed at enhancing the healing outcomes of periapical tissues following standardized endodontic treatment procedures, as well as inspire novel approaches to the treatment of apical periodontitis.

The discovery of cuproptosis provides new insights into the study of CAP. To elucidate the relationship between CAP and cuproptosis, we conducted bulk RNA sequencing on ten healthy apical periodontitis tissue samples and ten chronic apical periodontitis samples. Our analysis revealed a decrease in the expression of four CRGs in CAP: NFE2L2, DLST, SLC25A3, and GCSH. Conversely, the expression of four additional CRGs, namely NLRP3, ATP7A, ATP7B, and GLS, was found to be increased in CAP. Notably, NLRP3 and NFE2L2 have previously been associated with apical periodontitis (32, 33).

NLRP3 is primarily expressed in immune cells, where it detects pathogen- and damage-associated signals, thereby initiating inflammatory responses (34). The NLRP3 inflammasome complex is implicated in several pathways of cell death. Its activation is triggered by the release of oxidized mitochondrial DNA (mtDNA) and caspase-3/7-dependent potassium (K+) efflux (35). Reports indicate that NLRP3 plays a crucial role in the development and regulation of apical periodontitis (32). This involvement may lead to the degradation associated with apical periodontitis and bone destruction, consequently accelerating the progression of CAP. NFE2L2, commonly referred to as Nrf2, activates the expression of genes driven by antioxidant response elements that are involved in glutathione metabolism, detoxification enzymes, and antioxidant proteins, thus maintaining cellular redox homeostasis (36). It plays a crucial role in regulating the cellular response to oxidative and metal stress in various human diseases (37). Cells lacking NFE2L2 exhibit increased spontaneous apoptosis (38), and the dysfunction of NFE2L2 signaling within the periodontium may contribute to alveolar bone resorption (39). Additionally, NFE2L2 has been implicated in mitochondrial biogenesis disorders that promote refractory apical periodontitis (33). The dysfunction of NFE2L2 signaling in the periodontium could significantly influence alveolar bone resorption, potentially facilitating the progression of CAP (40).

The other six DE-CRGs have not been found to be associated with apical periodontitis; however, they have been implicated in various biological processes. GLS and DSLT have been reported to participate in the inflammatory process associated with periodontitis (41). Furthermore, GLS has been shown to be related to sensitivity to cell cuproptosis and is also involved in maintaining glutamate homeostasis (4). SLC25A3, functioning as the inner mitochondrial membrane phosphate transporter, has the capacity to transport copper from the intermembrane space of mitochondria across the inner membrane into the mitochondrial matrix. It has been reported to negatively regulate NLRP3 inflammasome activation in HEK293T cells (42). Additionally, ATPases, including ATP7A and ATP7B, are associated with the extracellular excretion of copper and export Cu ions bound to metal-binding sites in the presence of ATP (43). A mutation in ATP7A has resulted in decreased pro-inflammatory cytokine expression, suggesting its potential as a therapeutic target for inflammatory vascular disease (44).

We identified the diagnostic signatures associated with cuproptosis through logistic regression and subsequently established a diagnostic model. The 19 CRGs were selected to construct this model. Although there is insufficient evidence to suggest a strong association between these genes and CAP, they demonstrated a satisfactory diagnostic efficacy.

Additionally, we confirmed their expression levels in control and CAP tissues using qPCR. Our findings indicate that the expression changes of DE-CRGs align with the bioinformatics analysis of the entire CAP tissue, with the exception of NFE2L2 and SLC25A3. The observed discrepancies between experimental findings and bioinformatics predictions for these two genes may be attributed to sample heterogeneity. The pathogenesis and progression of CAP involve intricate interactions between inflammatory and anti-inflammatory mediators, which influence the process of CAP. Notably, dynamic variations in gene expression occur at different stages of apical periodontitis (45). NFE2L2, a master transcriptional regulator of cellular stress responses, is primarily governed by the KEAP1-Nrf2-ARE pathway. Under oxidative stress conditions, NFE2L2 becomes transiently activated to initiate antioxidant defenses; however, its activity is subsequently downregulated through self-regulatory mechanisms to establish negative feedback loops. This stress-responsive plasticity correlates closely with fluctuating NFE2L2 expression patterns (46). Regarding SLC25A3, this mitochondrial phosphate transporter may undergo dynamic modulation by inflammatory microenvironmental cues such as hypoxia and nutrient deprivation. While bioinformatics analyses capture static molecular “snapshots”, experimental samples likely represent heterogeneous metabolic states (47). Importantly, immunohistochemical validation revealed spatial expression patterns of both NFE2L2 and SLC25A3 in apical periodontitis lesions. Quantitative analysis demonstrated significantly upregulated expression compared to healthy controls, consistent with RT-PCR results.

Multiple cell types play a crucial role in balancing pro- and anti-inflammatory responses within the periapical microenvironment, which ultimately influences the progression and regression of CAP (48). Therefore, it is essential to assess the expression of DE-CRGs across the various cell types that constitute the CAP microenvironment. We conducted scRNA-seq analysis of CAP. Unlike conventional bulk sequencing, scRNA-seq enables the identification of specific cell clusters at the single-cell level (49). Our findings indicate that CAP induces alterations in the cellular composition of the apical periodontal tissue. Additionally, we examined the expression levels of CRGs across different cell types and observed significant differences in CRG expression among endothelial cells, fibroblasts, monocytes-macrophages, and dendritic cells. Notably, these four cell types also demonstrated an increased proportion in CAP. These results suggest that DE-CRGs may play a role in the establishment of CAP. Furthermore, we analyzed intercellular communication differences between various cell clusters in both CAP and healthy groups. The enhanced intercellular interactions between macrophages and fibroblasts in CAP underscore their role in connecting immune and non-immune cells, highlighting their significance in the pathogenesis of CAP.

We further selected fibroblasts and macrophages for a detailed analysis, classifying these two cell types into six distinct subgroups, respectively. Our findings indicate that the proportions of different subsets varied within CAP tissue. Further examination of the ligand-receptor interactions between macrophage and fibroblast subgroups revealed that macrophages engage with fibroblasts through pathways such as Notch signaling, which has been previously associated with copper (7). Notably, the largest proportions were observed in the COL4A1-Fibro and APOE-Macro subgroups within the CAP group. Collagen α-1 (IV) chain (COL4A1) serves as a critical component of the basement membrane across various tissues and cell types in the human body, playing a role in multiple pathological and physiological processes (50, 51). Apolipoprotein E (ApoE) is an essential constituent of lipoproteins, facilitating the binding of lipoproteins to receptors and contributing to the hepatic clearance of triglyceride-rich lipoproteins. As reported, macrophage expression of APOE reduces the expression of inflammatory cytokines and co-stimulatory molecules on the cell surface, thereby collectively limiting lesion inflammation (52–54).

The patterns of cell-cell communication observed in the COL4A1-Fibro and APOE-Macro subsets were both quantitatively and qualitatively significant. The results from GO and KEGG pathway analyses revealed that in CAP, COL4A1-Fibro exhibits strong enrichment in organelles and mitochondrial inner membrane components, suggesting a heightened susceptibility to copper-mediated mitochondrial toxicity. Given that cuproptosis is triggered by the copper-dependent aggregation of lipoylated TCA cycle proteins (e.g., DLAT, FDX1) (4), we propose that COL4A1-Fibro may serve as a primary site for copper accumulation and the initiation of cuproptosis in CAP. In contrast, APOE-Macro showed enrichment in GTPase activation and NTPase regulatory activity, indicating a role in regulating copper homeostasis. ApoE has been reported to increase intracellular copper levels by reducing the levels and delaying the trafficking of the copper transport protein, ATP7A, which may lead to impaired cellular copper export (55). GTPases regulate intracellular trafficking, lysosomal function, and mitochondrial dynamics, all of which are pertinent to copper metabolism and cuproptosis. Rab7, a small GTPase, controls lysosomal maturation and autophagy. It has been reported that mitochondria-lysosome contacts regulate mitochondrial fission via Rab7 GTP hydrolysis (56). Analysis of the expression of CRGs demonstrated differential expression between COL4A1-Fibro and APOE-Macro subsets. The differential expression of CRGs between these two cell subsets suggests cell-type-specific roles in cuproptosis. These findings indicate that CRGs are intricately related to the intercellular interactions between COL4A1-Fibro and APOE-Macro, thereby facilitating the onset and progression of cuproptosis in CAP.

**Figure 8:**
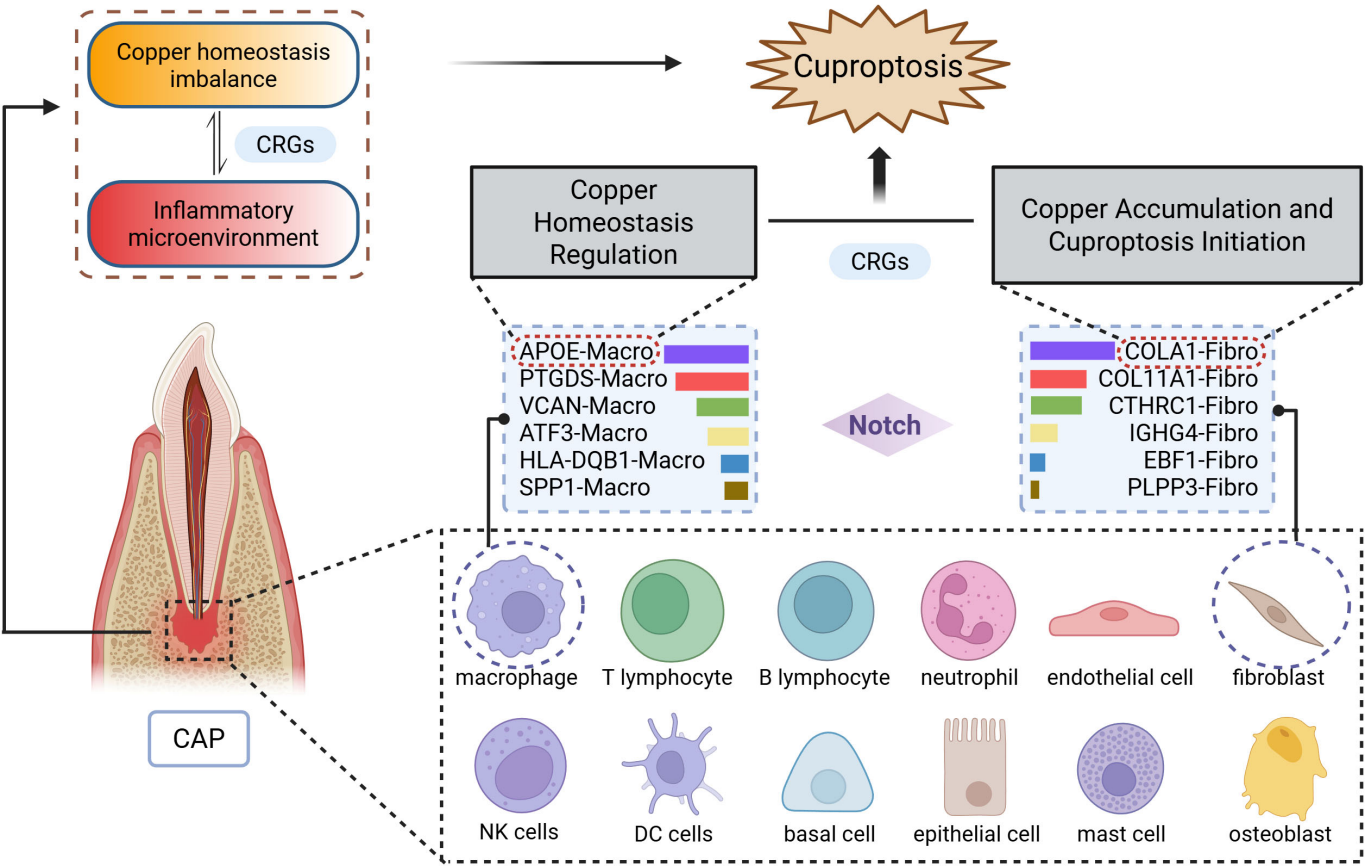
Schematic diagram illustrating the mechanism of cuproptosis in CAP. The CAP tissues consists of 12 major cell types, with particularly prominent interactions between macrophages (APOE-Macro subset) and fibroblasts (COL4A1-Fibro subset). Data were derived from single-cell RNA sequencing analysis.

As illustrated in **Figure 8**, single-cell RNA sequencing analysis elucidates the mechanism of cuproptosis in CAP tissues. Clinically, this research bridges an important gap between copper metabolism and CAP. AP typically develops as a sequel to untreated or inadequately treated pulpitis (13). It has been reported that LTA-induced pulpitis was alleviated when the cells or mice were treated with the copper chelator, tetrathiomolybdate (14). Our research identifies a gene signature that not only provides discriminative biomarkers but also reveals actionable therapeutic targets. Notably, the overlap between our top CRGs and the key regulators of cuproptosis (e.g., FDX1) (4) suggests translational potential, raising intriguing possibilities for the development of topical therapies.

In conclusion, we have identified CRGs associated with CAP for the first time through the integration of bulk and scRNA-seq, and we have further analyzed the involved cell clusters. This study suggests that cuproptosis plays a crucial role in the pathophysiological processes of CAP. Moreover, these findings provide important theoretical basis for developing novel CAP therapies targeting copper metabolism. However, we have to acknowledge that the current study has certain limitations, including a relatively limited sample size and substantial inter-individual heterogeneity. Additionally, targeted analyses are lacking, which hinders a more comprehensive understanding of the development of CAP and its underlying mechanisms. Future investigations will supplement clinical samples to enhance statistical power and conduct targeted analyses stratified by the severity of CAP.

## Data Availability

The datasets presented in this study can be found in online repositories. The names of the repository/repositories and accession number(s) can be found below: PRJNA1213680 (SRA).
