# Supplementary material for: Identification of cuproptosis-related genes in chronic apical periodontitis based on bulk and single-cell RNA sequencing analyses and experimental validation
Source: Front Immunol. 2025 Aug 20;16:1559220. doi: 10.3389/fimmu.2025.1559220 (PMC12404934; doi:10.3389/fimmu.2025.1559220)
Supplement: Supplementary file 2 [file DataSheet1.docx]

Supplementary Material

## Supplementary Figures

##
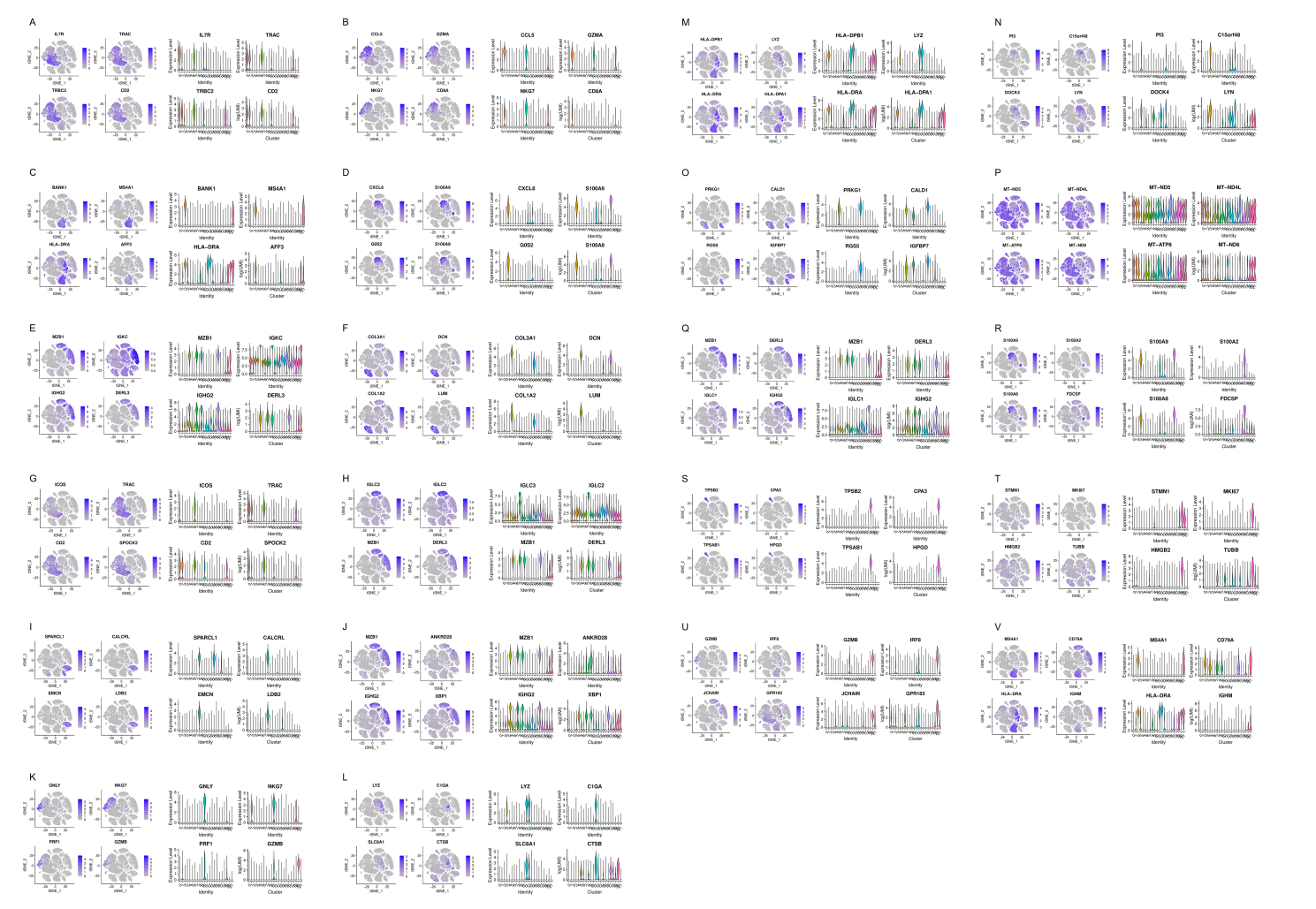


## **Supplementary Figure 1.** The expression levels of CRGs in the twelve primary cell clusters of the healthy control (HC) and chronic apical periodontitis (CAP) groups.


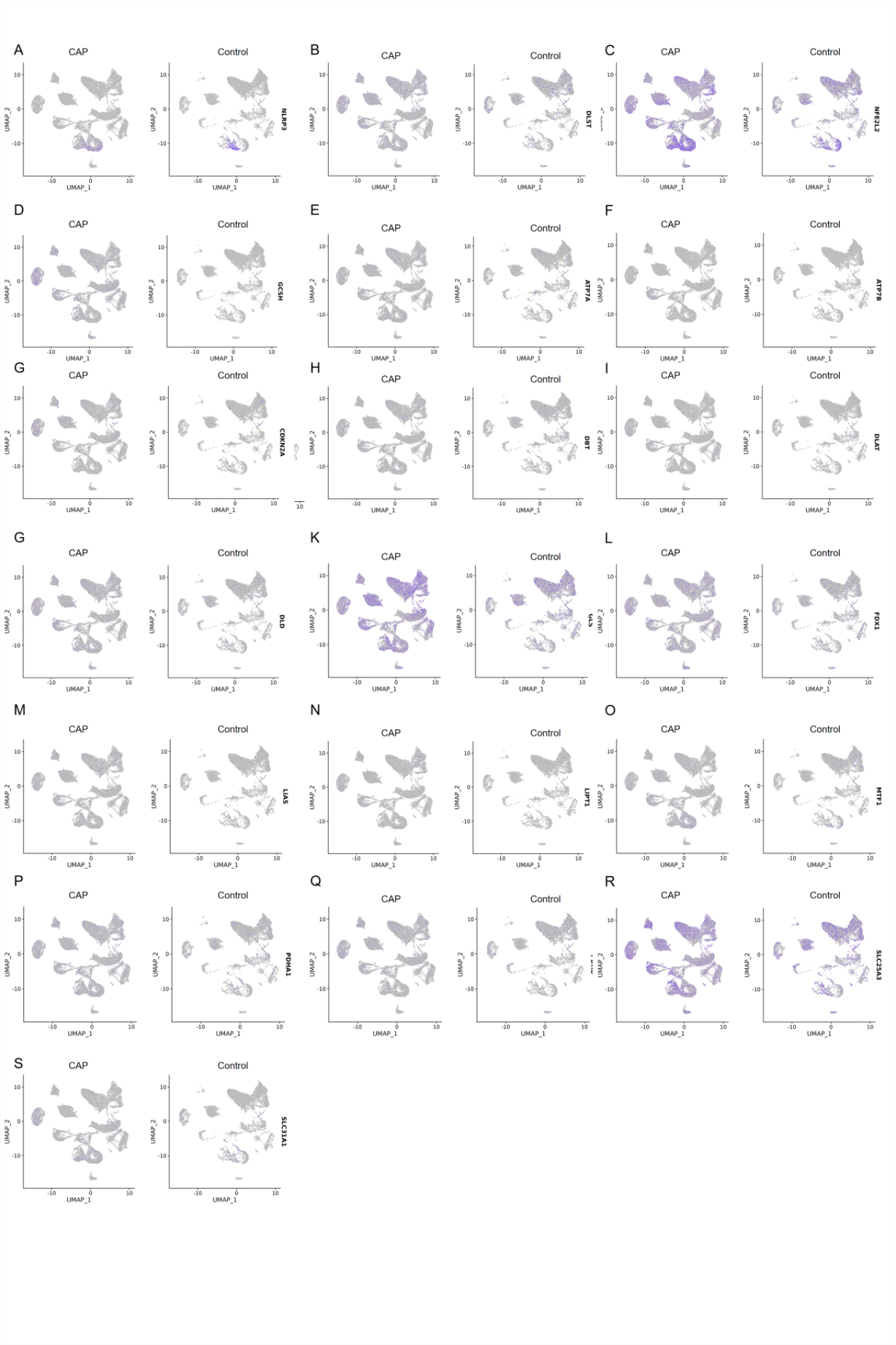


**Supplementary Figure 2.** Featureplot heatmap of DE-CRGs in CAP and Control. (A-S) Featureplot heatmap of each DE-CRGs in CAP and Control.


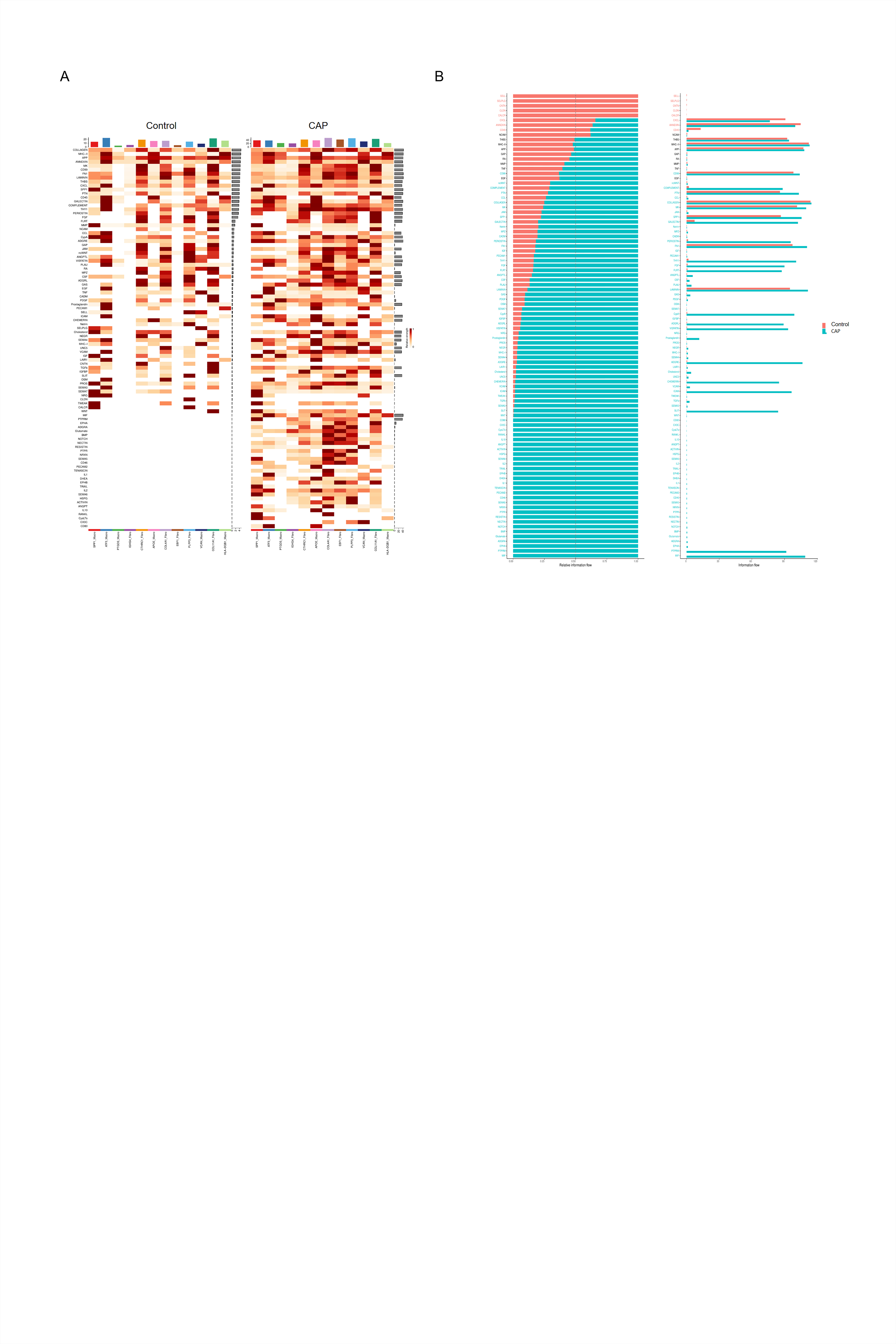


**Supplementary Figure 3.** (A) The signalingRole heatmap between CAP group and control group. (B) The barplot show the differential enrichment of signaling pathways between the CAP group and the control group.


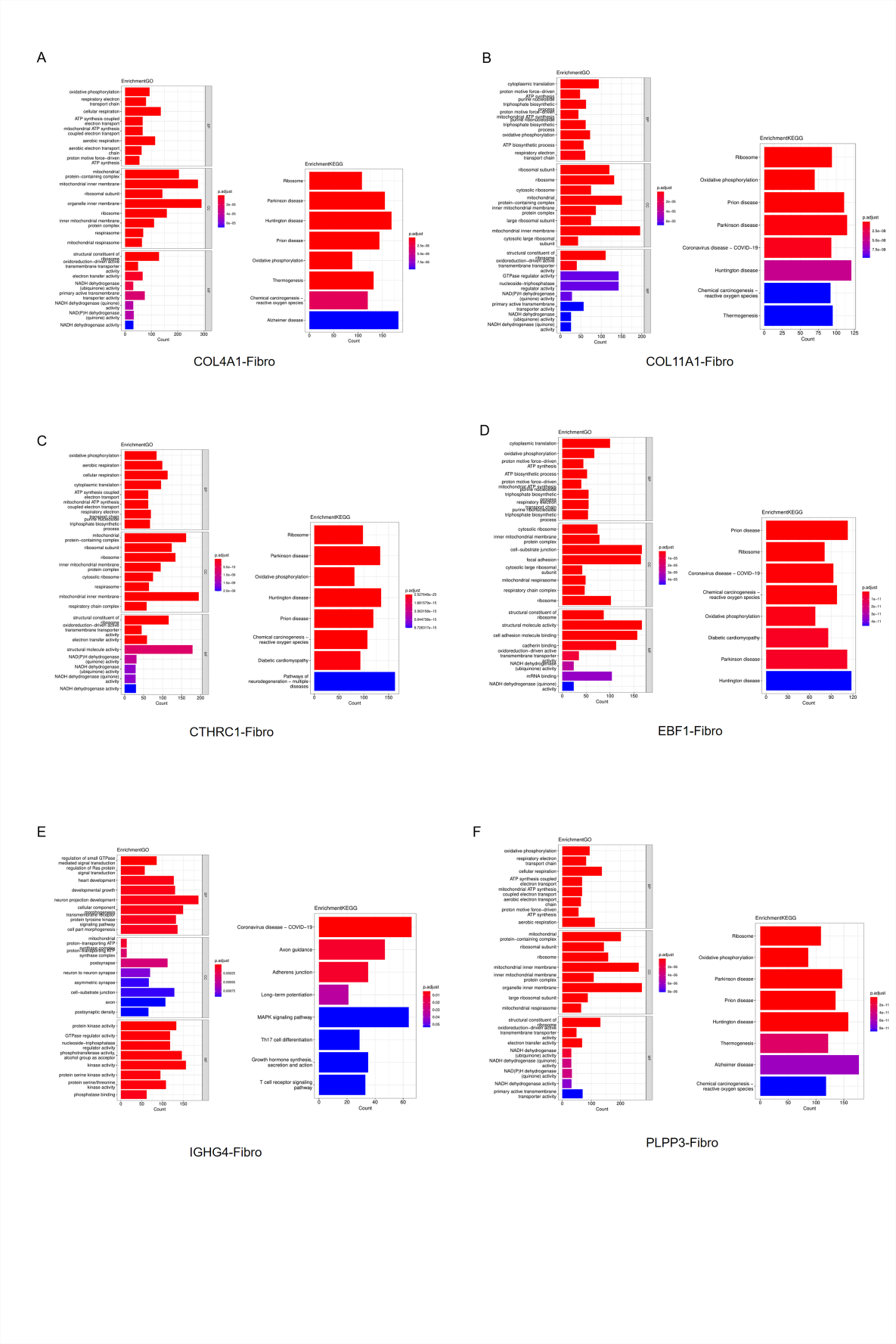


**Supplementary Figure 4.** Differential GO enrichment analysis and KEGG enrichment analysis of DEGs between CAP and control group in the fibroblast cell subsets. (A) COL4A1 fibroblast. (Fibro_1) (B) COL11A1 fibroblast. (Fibro_2) (C) CTHRC1 fibroblast. (Fibro_3) (D) EBF1 fibroblast. (Fibro_4) (E) IGHG4 fibroblast. (Fibro_5) (F) PLPP3 fibroblast. (Fibro_6)


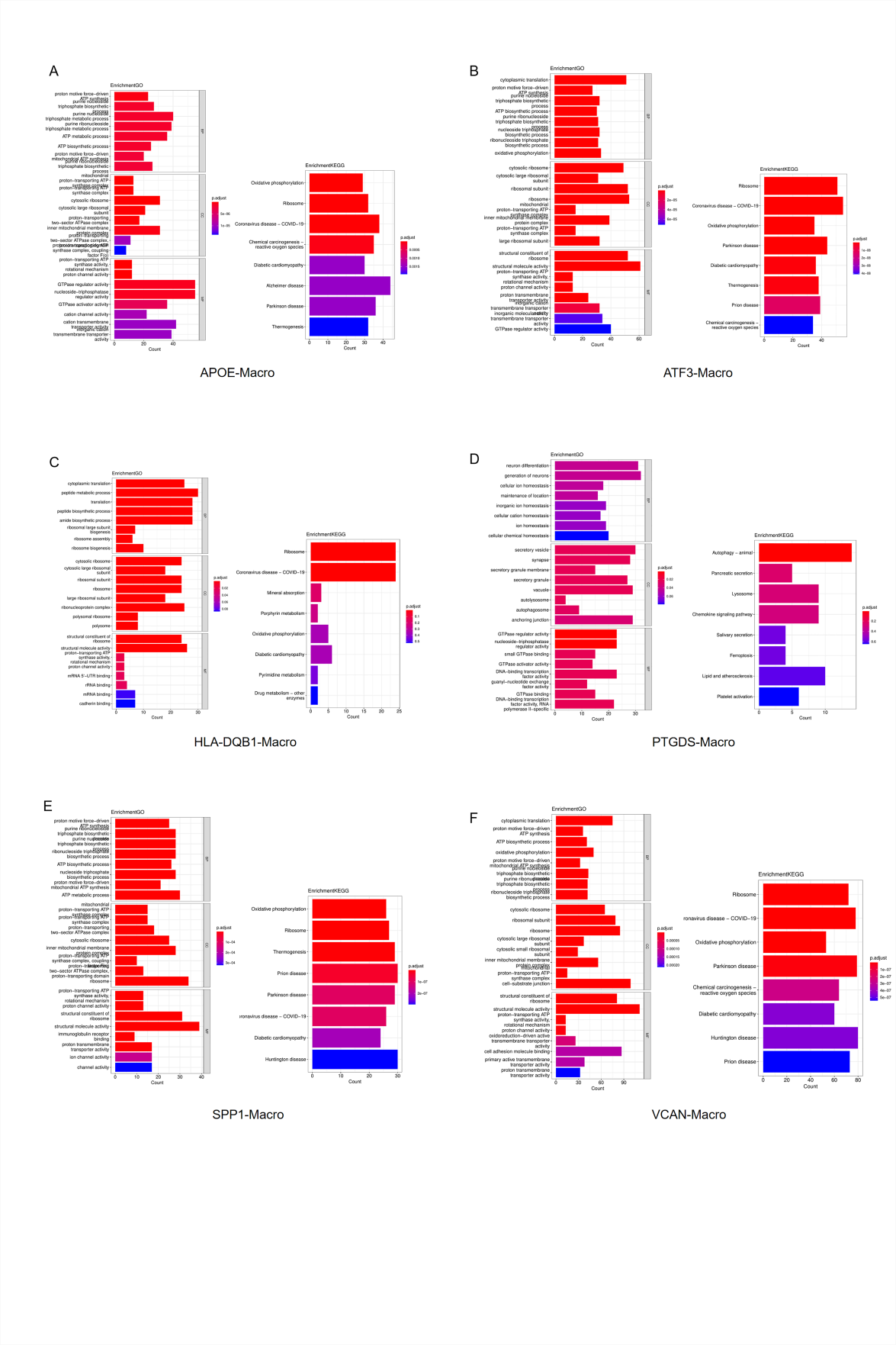


**Supplementary Figure 5.** Differential GO enrichment analysis and KEGG enrichment analysis of DEGs between CAP and control group in the fibroblast cell subsets. (A) COL4A1 fibroblast. (Fibro_1) (B) COL11A1 fibroblast. (Fibro_2) (C) CTHRC1 fibroblast. (Fibro_3) (D) EBF1 fibroblast. (Fibro_4) (E) IGHG4 fibroblast. (Fibro_5) (F) PLPP3 fibroblast. (Fibro_6)
