# Supplementary material for: Identification of cuproptosis-related genes in chronic apical periodontitis based on bulk and single-cell RNA sequencing analyses and experimental validation
Source: Front Immunol. 2025 Aug 20;16:1559220. doi: 10.3389/fimmu.2025.1559220 (PMC12404934; doi:10.3389/fimmu.2025.1559220)
Supplement: Supplementary file 3 [file DataSheet2.docx]

**Supplementary Data**


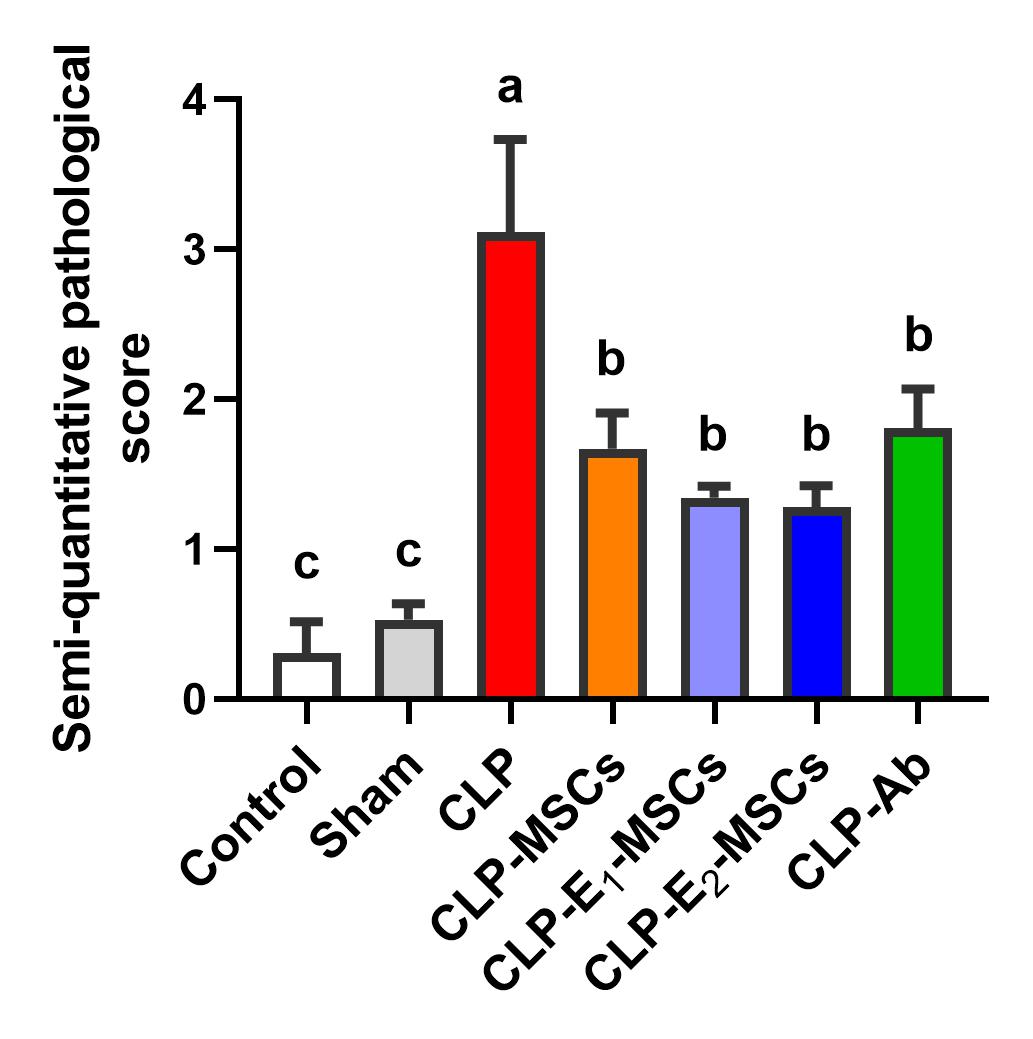


**Fig. S1.** Effects of mesenchymal stem cells (MSCs), mesenchymal stem cells enhanced with Na₂SeO₃ (E_1_-MSCs), mesenchymal stem cells enhanced with SeNPs (E_2_-MSCs), or standard treatment antibiotics (Ab) on the semi-quantitative analysis of the severity of every pathological alteration in the liver tissues of CLP-induced septic rats. The results are expressed as the mean ± SD of 10 fields. Different letters show statistically significant differences between groups by one-way ANOVA, with Tukey’s test at P < 0.05. Groups with the same letter have no significant differences.
